# Congenital Anomalies in Canada Data Exploration Tool: latest update on prevalence estimates and temporal trends for congenital anomalies over 15 years (2008–2023)

**DOI:** 10.24095/hpcdp.45.6.05

**Published:** 2025-06

**Authors:** Chantal Nelson, Katarzyna Naczk, Neetu Shukla, Yuan Xu, Parnian Hossein-Pour, Hongbo Liang, Catherine Pelletier

**Affiliations:** 1 Lifespan Chronic Disease and Conditions Division, Health Promotion and Chronic Disease Prevention Branch, Public Health Agency of Canada, Ottawa, Ontario, Canada; 2 Surveillance Systems and Data Management Division, Health Promotion and Chronic Disease Prevention Branch, Public Health Agency of Canada, Ottawa, Ontario, Canada

The Public Health Agency of Canada (PHAC) is pleased to announce the release of the latest Congenital Anomalies in Canada Data Exploration Tool. The interactive Data Exploration Tool, located on the Health Infobase website (https://health-infobase.canada.ca/congenital-anomalies/), has recent information on 38 congenital anomalies grouped into 12 categories based on the Canadian modiﬁcationof the *International Classification of Diseases and Related Health Problems, Tenth Revision* (ICD-10-CA). 

The data shown in the Data Exploration Tool were derived from the Canadian Institute for Health Information’s Discharge Abstract Database, as well as the Maintenance et exploitation des donnes pour l’tude de la clientle hospitalire (MED-CHO) database. The Data Tool shows prevalence estimates and temporal trends of congenital anomalies at the national and jurisdictional level (excluding Alberta). The data include livebirth and stillbirth data from 2008 to 2023 using a follow-up period of up to 1 year after birth. Data from Quebec include livebirth data from 2008 to 2022.

This edition of the Congenital Anomalies Data Exploration Tool includes a new “Quick Facts” page, a new “Supplemental Congenital Anomalies Statistics” section, and a new “Additional Resources” tab.

Notable findings include:

- Temporal trends for most congenital anomaly categories remained stable at the national level over the years 2008 to 2023; however, there was an increasing trend for urinary tract defects and a decreasing trend for central nervous system defects.
- Case fatality rates were highest among infants with neural tube defects (24.1 per 100 livebirths), with the highest fatality rates among infants with anencephaly (86.0 per 100 livebirths).

